# Management of anterior and posterior crossbites with lingual appliances and miniscrew-assisted rapid palatal expansion: A case report

**DOI:** 10.1097/MD.0000000000040832

**Published:** 2024-12-06

**Authors:** Viet Anh Nguyen, Ngoc Anh Nguyen, Hong Le Doan, Thi Hanh Pham, Bich Ngoc Doan

**Affiliations:** a Faculty of Dentistry, Phenikaa University, Hanoi, Vietnam; b Private Practice, Viet Anh Orthodontic Clinic, Hanoi, Vietnam.

**Keywords:** class III malocclusion, lingual appliances, MARPE, posterior crossbite, rapid palatal expansion

## Abstract

**Rationale:**

Current literature describes only 2 cases combining miniscrew-assisted rapid palatal expansion (MARPE) with lingual appliances. These cases require 2 impressions, 1 before and 1 after palatal expansion, to ensure accurate lingual appliance placement, potentially increasing treatment time and cost. This case report aimed to demonstrate a modified workflow of combining MARPE and lingual appliances in managing an adult patient with anteroposterior and transversal discrepancies, requiring only a single digital impression for both fabrication and positioning.

**Patient concerns:**

A 29-year-old female presented with anterior and posterior crossbites, a class III skeletal relationship, and maxillary constriction. The patient desired a nonsurgical and esthetic treatment approach.

**Diagnoses:**

The patient was diagnosed with a class III malocclusion with anterior and posterior crossbite on a skeletal hypodivergent class III relationship and upper posterior constriction.

**Interventions:**

Treatment included MARPE for skeletal expansion, digitally planned lingual appliances for tooth alignment, and lower arch distalization for class III correction. A modified appliance sequence was utilized for optimal expansion retention and lingual bracket transfer accuracy.

**Outcomes:**

The treatment successfully corrected the crossbites, improved dental and facial esthetics, and achieved partial skeletal correction.

**Lessons:**

The combination of MARPE and lingual appliances offers a viable treatment alternative that prioritizes both esthetics and effective skeletal expansion for nonsurgical and esthetic management of adult class III malocclusion with maxillary constriction. The modified workflow, employing a single digital impression, may offer several advantages, including reduced treatment duration and associated costs, prolonged postexpansion retention, and minimized aesthetic impact of midline diastema.

## 1. Introduction

Crossbites are common orthodontic problems with possible impacts on oral health, facial aesthetics, and jaw function.^[1]^ Anterior crossbites can lead to dental attrition and gingival recession, while posterior crossbites can cause functional shifts of the mandible and temporomandibular joint disorders.^[2]^ Therefore, early diagnosis and appropriate corrective management are crucial for optimal long-term outcomes. Treatment alternatives for anterior crossbites include upper incisor protraction or lower incisor retraction. On the other hand, posterior crossbites can be managed with maxillary arch expansion or mandibular arch constriction.

Classically, maxillary arch expansion in adult patients generally involves dental compensation due to fused midpalatal sutures. Dental expansion can be achieved with a removable expansion plate, quad-helix, and rapid palatal expander. The disadvantages of dental compensation include buccal tipping of maxillary molar crowns, increased risk of periodontal breakdown, and high relapse tendency. Surgically assisted rapid palatal expansion and maxillary segmental osteotomy were previously the only options for skeletal expansion in adults.^[3]^ However, their invasive nature may deter many patients. The introduction of miniscrew-assisted rapid palatal expansion (MARPE) offers a nonsurgical alternative for skeletal expansion in adults.^[4]^ However, most articles in the literature present the combination of MARPE and regular labial fixed appliances.^[5]^

Lingual appliances are preferred to labial ones in many adult patients with high esthetic demands during orthodontic treatment.^[6]^ Labial appliances can be visible during smiles, possibly affecting the self-confidence of such adult patients. In adult patients with maxillary transverse deficiency, lingual appliances combined with MARPE become a viable nonsurgical esthetic treatment option.^[7]^ However, first molar bands may interfere with lingual bracket bonding as lingual brackets are generally indirectly bonded based on an ideal orthodontic setup, unlike labial ones. Therefore, a modified treatment sequence is necessary when combining MARPE with lingual appliances. While current literature describes only 2 cases combining MARPE and lingual appliances for adults with maxillary constriction, their approach has limitations.^[7,8]^ These cases required 2 impressions, 1 before and 1 after palatal expansion, to ensure accurate lingual appliance placement, potentially increasing treatment time and cost.

This case report aimed to present the management of an adult patient with skeletal class III malocclusion, anterior, and posterior crossbites. The treatment strategy included MARPE and digitally planned straight-wire lingual appliances, requiring only a single digital impression for both fabrication and positioning.

## 2. Case presentation

### 2.1. Diagnosis and etiology

A 29-year-old female patient presented with chief complaints of an anterior crossbite and prominent lower lip. The patient had not received any prior orthodontic treatment. Her medical and dental histories were noncontributory.

**Figure 1.**
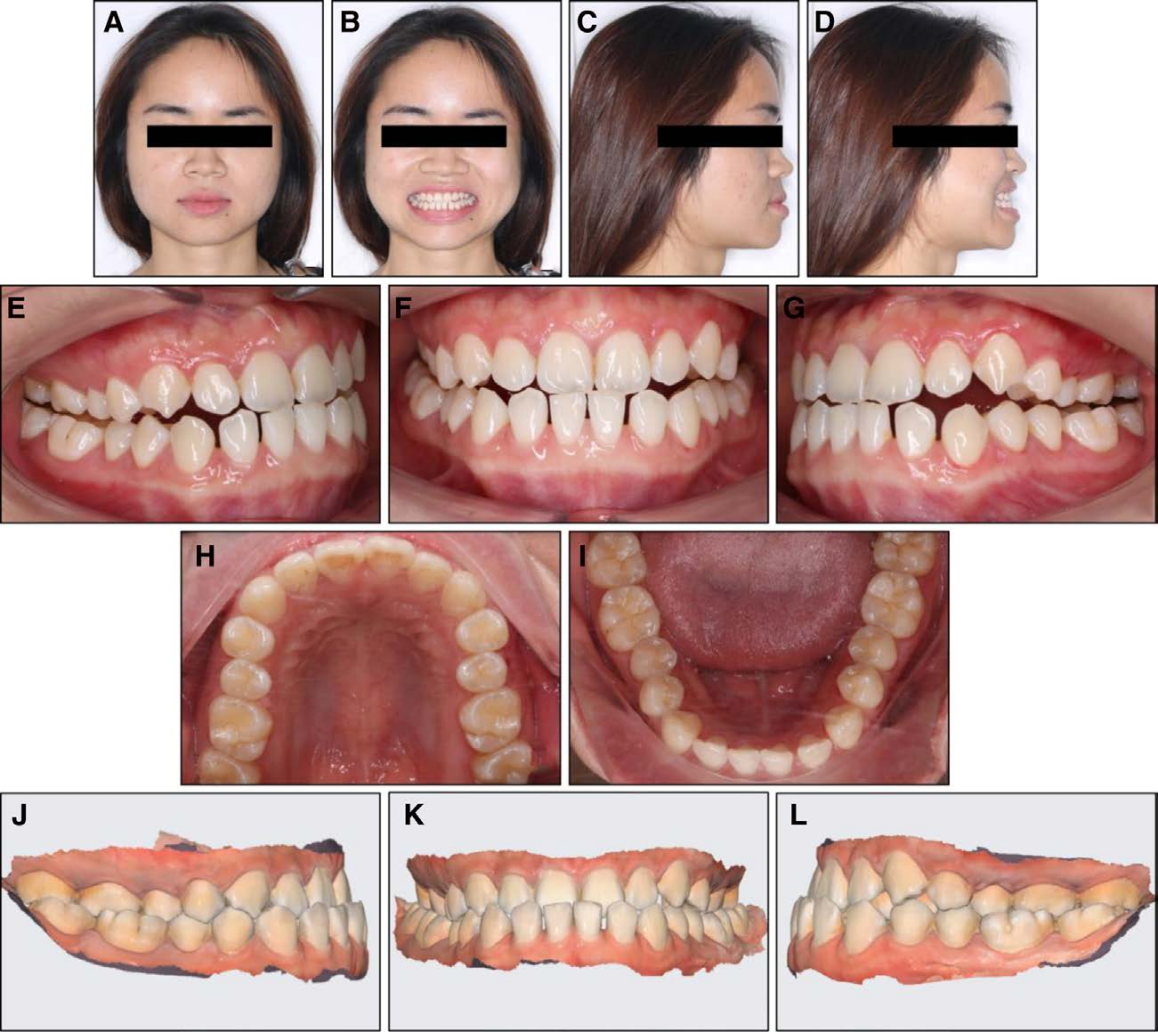
Pretreatment extraoral and intraoral photographs: (A) frontal, (B) frontal smiling, (C) lateral, (D) lateral smiling, (E) right occlusion in centric relation, (F) anterior occlusion in centric relation, (G) left occlusion in centric relation, (H) upper arch, (I) lower arch, (J) right occlusion in maximal intercuspal position, (K) anterior occlusion in maximal intercuspal position, (L) left occlusion in maximal intercuspal position.

On an extraoral examination, the patient showed a balanced vertical proportion of her face (Fig. 1). Her profile was slightly convex with her lower lip lying in front of her upper lip. The patient’s mandible deviated to the right side. No signs of a temporomandibular joint disorder were recorded.

On an intraoral examination, the patient exhibited anterior and posterior crossbites with a negative overjet of −1.6 mm. She had class III canine and molar relationships on both sides. Additionally, the patient had a constricted upper inter-molar width of 49.4 mm.^[9]^ The patient showed mild crowding of 1.8 mm in the upper arch and spacing of 5.2 mm in the lower arch. The upper dental midline was coincident with the facial midline, while the lower dental midline deviated 1.8 mm to the right side.

**Figure 2.**
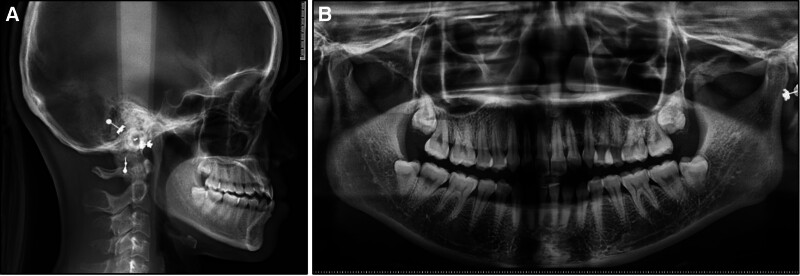
Pretreatment radiographs: (A) lateral cephalometric radiograph, (B) panoramic radiograph.

On the panoramic radiograph, all teeth were present including the third molars (Fig. 2). A lateral cephalometric evaluation showed a skeletal class III relationship (point A-nasion-point B: −1.7°; wits appraisal: −4.1 mm) and a hypodivergent facial pattern (Frankfort mandibular angle: 19.6°) (Table 1). The upper incisors had a normal inclination (upper incisor to sella-nasion: 106.1°) while the lower incisors were slightly proclined (incisor mandibular plane angle: 93.7°). The lower lip was protruded (lower lip/E-line: 2.1 mm).

**Table 1:** Lateral cephalometric and transversal arch measurements.

Measurements	Pretreatment	Posttreatment	Norm
Skeletal			
SNA (°)	79.1	79.3	81.1 ± 3.7
SNB (°)	80.8	80.5	79.2 ± 3.8
ANB (°)	−1.7	−1.2	2.5 ± 1.8
FMA (°)	19.6	19.3	25.0 ± 4.0
Wits appraisal (mm)	−4.1	−2.8	0.4 ± 2.3
Dental			
Upper incisor/SN (°)	106.1	105.1	105.3 ± 6.6
Upper incisor/NA (°)	26.9	25.3	22.0 ± 5.0
Upper incisor/NA (mm)	5.4	5.9	4.0 ± 3.0
IMPA (°)	93.6	87.5	90.0 ± 3.5
Lower incisor/NB (°)	24.0	18.1	25.0 ± 5.0
Lower incisor/NB (mm)	4.9	2.6	4.0 ± 2.0
Interincisal angle (°)	130.8	138.2	128.0 ± 5.3
Overjet (mm)	−1.6	1.8	2.0 ± 2.0
Overbite (mm)	2.4	1.6	2.0 ± 2.0
Upper inter-canine width (mm)	36.5	36.8	35.1 ± 0.3
Lower inter-canine width (mm)	30.9	29.1	27.3 ± 0.2
Upper inter-molar width (mm)	49.4	54.5	52.6 ± 0.2
Lower inter-molar width (mm)	50.0	50.2	44.9 ± 0.2
Soft tissue			
Upper lip/E-line (mm)	−1.0	−0.9	0.0 ± 2.0
Lower lip/E-line (mm)	2.1	1.8	0.0 ± 2.0

ANB = point A-nasion-point B, FMA = Frankfort mandibular angle, IMPA = incisor mandibular plane angle, NA = nasion-point A, NB = nasion-point B, SN = sella-nasion, SNA = sella-nasion-point A, SNB = sella-nasion-point B.

The patient was diagnosed with a dental class III malocclusion on a skeletal class III relationship, hypodivergent facial pattern, right deviated mandible, upper posterior constriction, slight upper arch crowding, lower arch spacing, and slightly proclined lower incisors.

### 2.2. Treatment plan

The patient denied any type of surgical intervention. She also desired invisible appliances without the need to remove them during meals. Therefore, fixed lingual appliances were used in combination with maxillary skeletal expansion using MARPE. Additionally, miniscrew-assisted distalization of the lower arch was planned to correct the class III relationship and anterior crossbite. The third molars would be extracted to allow the distalization of the lower second molars.

### 2.3. Treatment progress

A digital impression of the patient’s dentition was taken with an intraoral scanner (i500, Medit, Korea) and exported as standard triangular language files. The standard triangular language files were imported into orthodontic software (Autolign, Diorco, Korea) to create an ideal orthodontic setup with aligned teeth, a class I dental relationship, normal overjet, and overbite. Virtual lingual bracket placement was performed based on this setup and straight lingual arch-forms. The study models with lingual virtual brackets were exported from the software and printed with a resin 3-dimensional (3D) printer (Saturn S, Elegoo, Shenzhen, China). Double vacuum-formed indirect bonding trays were made with these 3D-printed models.^[6,10]^ Additionally, a MARPE (A0620, Leone, Italy) was fabricated by an orthodontic technician.

**Figure 3.**
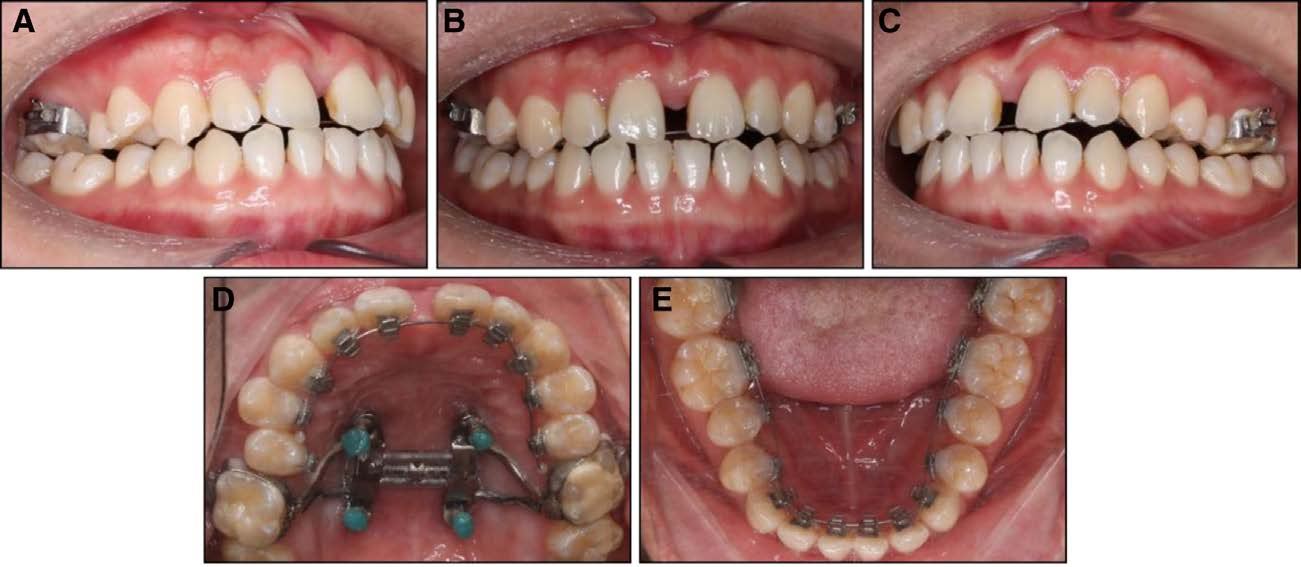
Midpalatal suture opening indicated by the upper midline diastema: (A) right occlusion, (B) anterior occlusion, (C) left occlusion, (D) upper arch, (E) lower arch.

Treatment commenced by bonding all teeth with 0.018” × 0.025” lingual appliances (ADB, Medico, Korea) except the upper first molars. The MARPE was placed with 2 larger anterior miniscrews (diameter: 2.0 mm; length: 12 mm; Hi-fix, Medico, Korea) and 2 smaller posterior miniscrews (diameter: 1.6 mm, length: 10 mm) on the same day of bonding lingual appliances. The expander was activated at the rate of 2 turns equal to 0.5 mm per day. 2 weeks later, the expansion was completed with a large diastema between the upper central incisor indicating midpalatal suture opening (Fig. 3).

After 1 month of treatment, lingual straight archwires were engaged into the lingual brackets with the sequence of 0.014,” 0.016,” 0.016” × 0.022” nickel-titanium, and 0.016 × 0.022 stainless steel archwires in both arches. After 2 months of treatment, MARPE’s arms were sectioned to replace the first molar bands with lingual brackets indirectly bonded with individual transfer jigs.^[11]^ The MARPE was still left in place to maintain the expansion.

**Figure 4.**
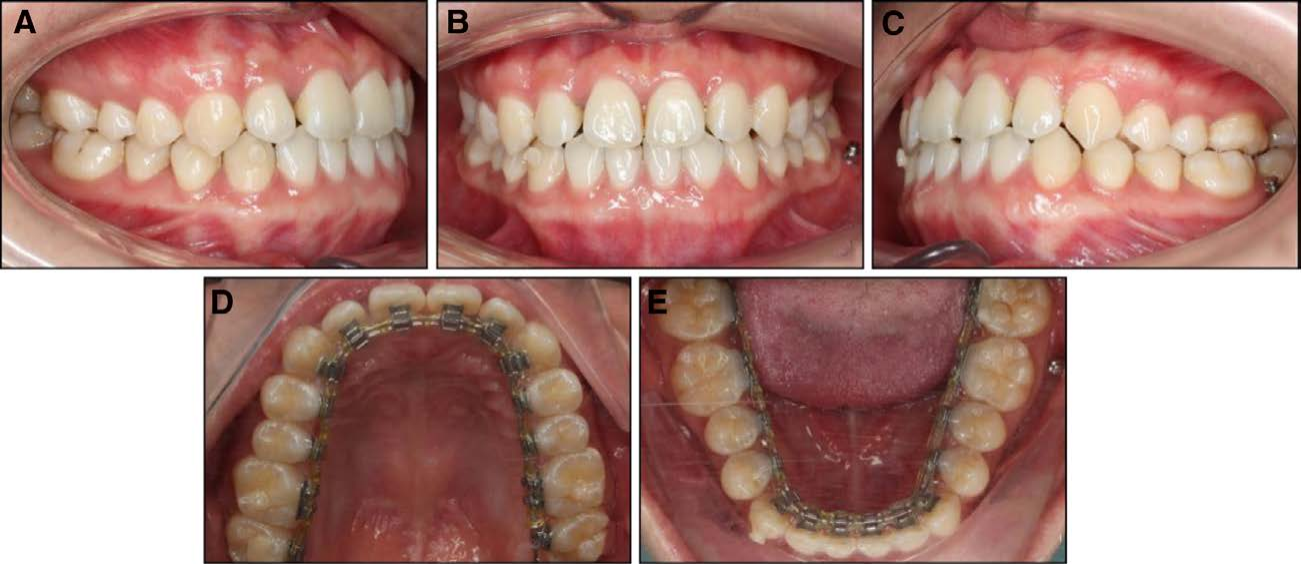
Intraoral photographs in the 10th mo: (A) right occlusion, (B) anterior occlusion, (C) left occlusion, (D) upper arch, (E) lower arch.

After 6 months of treatment, the MARPE was removed. Two miniscrews (diameter: 2.0 mm; length: 12 mm) were inserted into mandibular buccal shelves to apply distalizing forces of 200 g per side to the entire lower arch. One month later, the distalizing forces were discontinued on the right side and maintained on the left side to correct the lower dental midline deviation. The distalization stage took 3 months (Fig. 4). The total active treatment time was 11 months. After lingual appliance removal, lingual fixed retainers were bonded in both arches. The patient was satisfied with the treatment outcomes and the invisible nature of lingual appliances with only minimal discomfort during the first 2 weeks after appliance placement.

### 2.4. Treatment results

**Figure 5.**
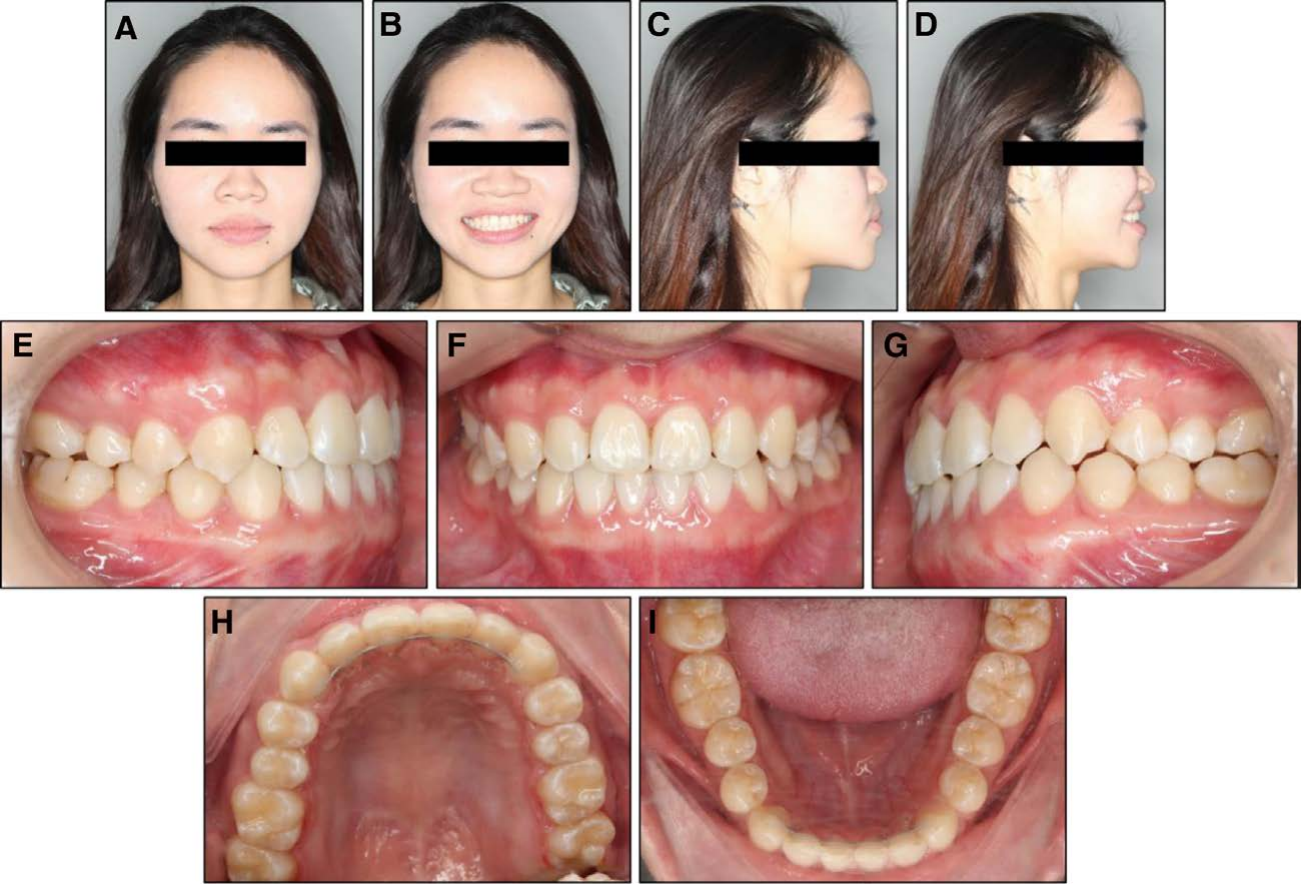
Posttreatment extraoral and intraoral photographs: (A) frontal, (B) frontal smiling, (C) lateral, (D) lateral smiling, (E) right occlusion, (F) anterior occlusion, (G) left occlusion, (H) upper arch, (I) lower arch.

Posttreatment records showed an improvement in dentofacial esthetics and functional occlusion (Fig. 5). Class I canine and molar relationships were obtained on both sides with a normal overbite and positive overjet. Both arches were well-aligned with all spaces closed. The upper inter-molar width was expanded by 5.1 mm. The lower dental midline, previously deviated to the right, was now centered with both the upper and facial midlines.

**Figure 6.**
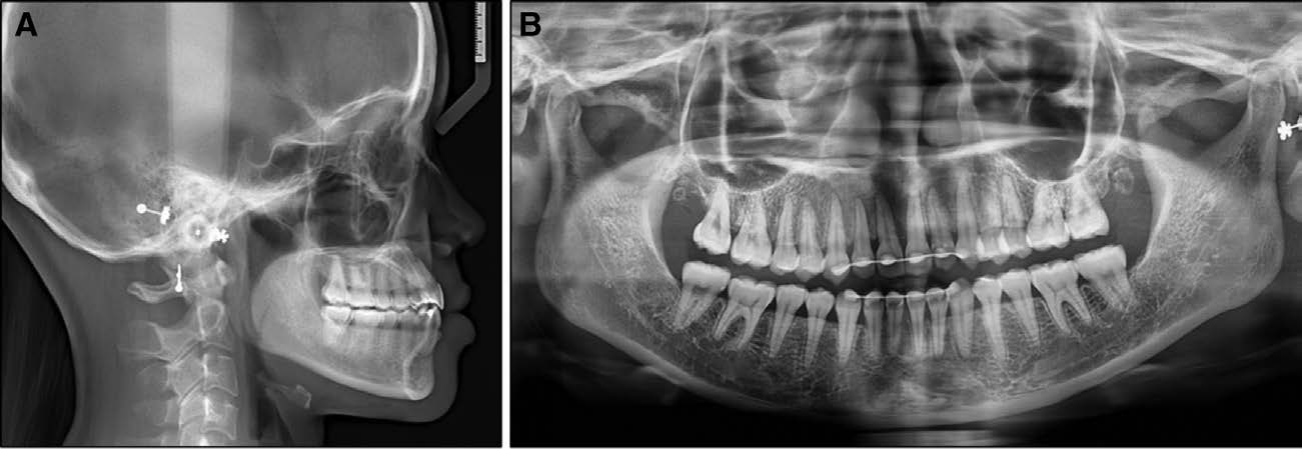
Posttreatment radiographs: (A) lateral cephalometric radiograph, (B) panoramic radiograph.

**Figure 7.**
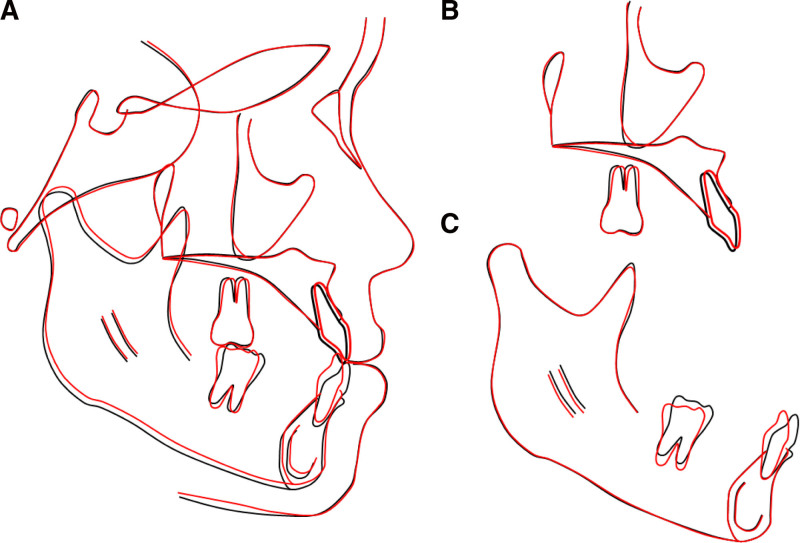
Lateral cephalometric superimpositions, before (black) and after treatment (red): (A) overall superimposition, (B) maxillary superimposition, (C) mandibular superimposition.

A posttreatment radiograph assessment demonstrated adequate root parallelism and no signs of root resorption (Fig. 6). A posttreatment lateral cephalometric evaluation showed an improvement in the skeletal class III relationship (point A-nasion-point B: −1.2°; wits appraisal: −2.8 mm) and lower incisor inclination (incisor mandibular plane angle: 87.5°). Lateral cephalometric superimpositions indicated the distalization of the lower molars and incisors (Fig. 7).

**Figure 8.**
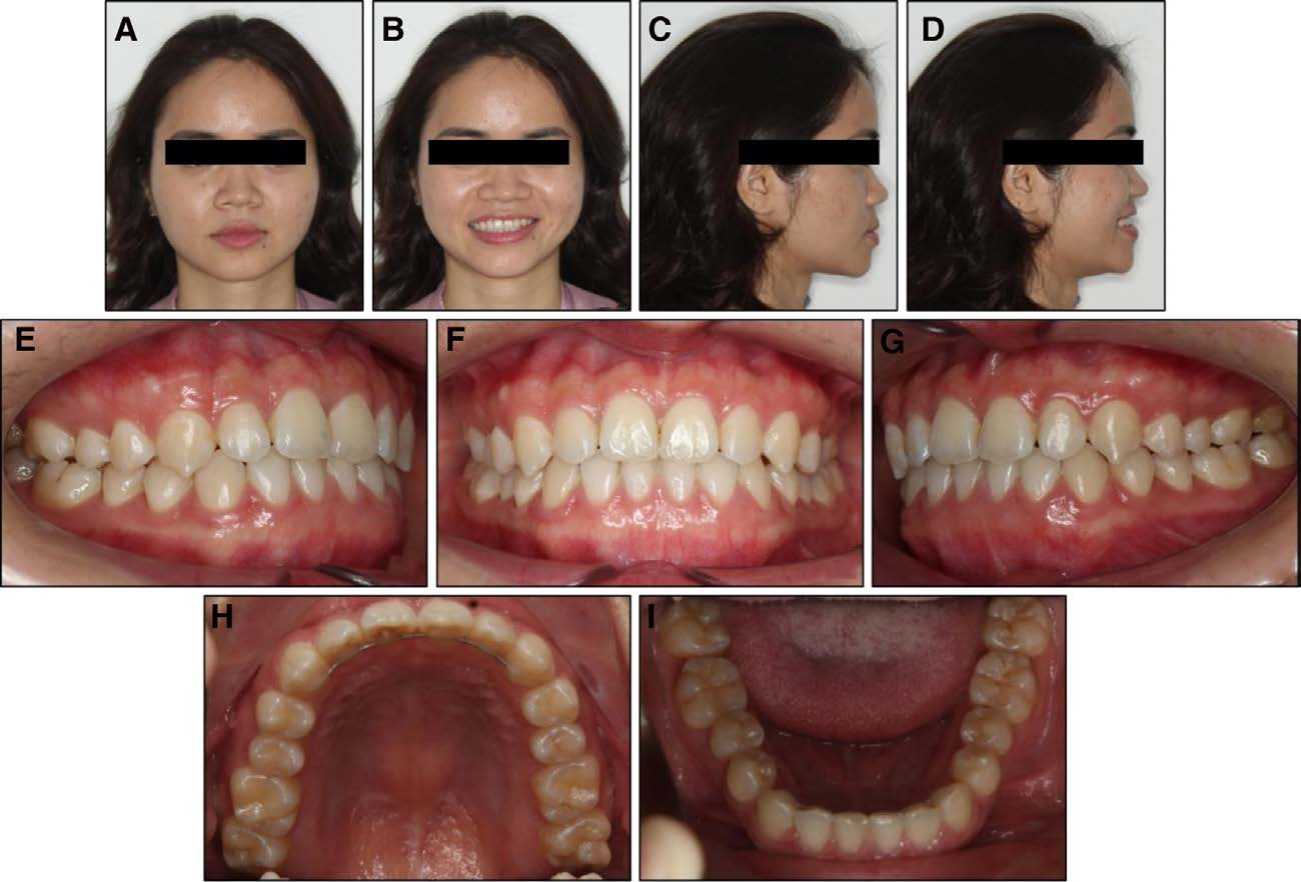
One-year post-retention extraoral and intraoral photographs: (A) frontal, (B) frontal smiling, (C) lateral, (D) lateral smiling, (E) right occlusion, (F) anterior occlusion, (G) left occlusion, (H) upper arch, (I) lower arch.

The patient was reevaluated 1-year following appliance removal. The 1-year post-retention record demonstrated the treatment result to be stable without signs of relapse (Fig. 8).

## 3. Discussion

This case demonstrates the efficacy of combining lingual appliances with MARPE for nonsurgical treatment of adult patients with skeletal class III malocclusion and maxillary constriction. The use of lingual appliances highlights a patient-centered approach that prioritizes esthetics during orthodontic management. Additionally, MARPE effectively achieved the necessary skeletal expansion, avoiding a more invasive surgical intervention. On the other hand, skeletal expansion overcomes the disadvantages of dental compensation including thinning of the buccal alveolar wall and the subsequent bony dehiscence formation. Additionally, the molar crown tipping is more limited with skeletal expansion compared to dental compensation, which could offer more stable results.

In this patient, mid-treatment records indicated near-complete correction of the mandibular lateral deviation following palatal expansion. This improvement likely resulted from the removal of functional interferences. Dental compensation alone might not have achieved this outcome, as it could have caused upper molar tipping and subsequent occlusal disturbances related to the extrusion of the maxillary molars’ palatal cusps.

Although MARPE successfully opens the midpalatal suture, resistance from surrounding circum-maxillary structures persists. This resistance can cause undesirable dentoalveolar tipping and expander deformation. While MARPE doesn’t fully overcome this resistance like Surgically assisted rapid palatal expansion, further investigations are necessary to directly compare the effectiveness of these 2 approaches. Cremonini et al^[8]^ proposed a tricortical anchorage system involving the palate, maxillary sinus, and nasal floor to increase primary stability for more efficient opening of the midpalatal suture. However, this approach is complicated, requiring cone beam computed tomography for planning which can expose patients to unnecessary additional radiation. Furthermore, a 3D surgical guide is required to insert miniscrews, possibly reducing the cost-effectiveness. Additionally, the increased bulkiness of the appliance may lead to patient discomfort.

Direct bonding of labial brackets simplifies the combination of MARPE and traditional labial appliances, as it eliminates the need for a second impression and indirect bonding tray fabrication.^[4,5]^ However, combining MARPE with lingual appliances presents a challenge due to the indirect bonding of lingual brackets. Pretreatment impressions cannot be used for fabricating indirect bonding trays because tooth positions change after expansion with MARPE. Furthermore, MARPE usually requires banding the upper first molars, which prevents bonding brackets on all teeth before MARPE placement.

Takagi and Tanaka^[7]^ employed a technique where impressions for lingual bracket bonding were taken after the expansion and retention stages. Unfortunately, this strategy resulted in a persistent diastema between the upper central incisors. This space, which remained for 3 to 6 months of retention, could potentially have a significant negative impact on a patient’s self-confidence. Consequently, shortening the retention period might be necessary but could compromise the stability of the achieved expansion. Additionally, tooth movement after MARPE removal might occur, potentially reducing the accuracy of transferring bracket positioning during indirect bonding. To address this, we performed the lingual indirect bonding procedures before MARPE insertion, bypassing the first molars. After an initial retention period, MARPE arms were sectioned, allowing for individual bonding of those teeth without the need for full MARPE removal.

Similar to reports by Takagi et al, Cremonini et al, and Fukawa et al this case demonstrates successful nonsurgical skeletal expansion in an adult using MARPE with lingual appliances.^[7,8,12]^ However, mushroom archwires and analog indirect bonding procedures were used in these previous studies instead of digitally-planned straight archwire lingual appliances. The use of straight archwires in lingual orthodontics can make the archwire forming process easier compared to traditional mushroom archwires.^[11,13]^ Additionally, the application of digital technology in lingual orthodontic treatment planning can simplify the orthodontic setup creation and indirect lingual bracket bonding procedures, making in-office workflows possible.^[11,14]^ Arveda et al^[15]^ combined MARPE with sectional lingual appliances and clear aligners, in which lingual appliances were used to correct severely rotated teeth and close the diastema resulting from skeletal expansion. However, the use of multiple appliance types might increase treatment complexity and lead to additional costs. The introduction of digital MARPE may further enhance workflows for combining MARPE with digital fixed appliances.^[16]^

The use of the lingual straight-wire technique offers several advantages, including avoiding time-consuming wire bending and simplifying the treatment procedure, which is particularly beneficial in lingual orthodontics due to limited working fields and unfavorable posture. Sliding mechanics can also be used smoothly without interferences of the offset bends with lingual brackets. Finally, the straight-wire technique facilitates the placement of finishing and detailing bends without interfering with the offset bends, further improving the precision and efficiency of lingual orthodontic treatment.^[17]^

The retention of the expanded transversal arch width has an important effect on the stability of the treatment results, especially in cases with major expansions.^[18]^ Therefore, lingual fixed retainers were placed in the anterior segments in both arches for long-term stability. Furthermore, the ability to simultaneously place lingual appliances with MARPE allowed for longer intraoral retention of the expander, promoting palatal suture bone formation and potentially improving stability.

A primary limitation of this study is its case report design, which may limit the generalizability of the treatment combination’s effectiveness to a larger population. Additionally, the study employed a partially analog workflow for fabricating MARPE, as opposed to a fully digital workflow with 3D-printed palatal bands and arms. This choice was made because fully digital workflows often necessitate welding 3D-printed components to the expander, potentially affecting its performance and increasing resistance during activation.^[16]^

## 4. Conclusions

This case illustrates the potential of combining MARPE and lingual appliances for invisible and efficient treatment of adult patients with class III malocclusion and transversal discrepancy. This highlights a viable treatment alternative that prioritizes both esthetics and effective skeletal expansion. The modified workflow, using a single digital impression, may reduce treatment time and costs while allowing for longer post-expansion retention. Additionally, this approach minimizes aesthetic concerns related to midline diastema. Further studies are needed to confirm the effectiveness and efficiency of the approach.

## Acknowledgments

The authors express their sincere gratitude to the patient for their consent to publish their clinical photographs and radiographs in this case report.

## Author contributions

**Conceptualization:** Viet Anh Nguyen, Ngoc Anh Nguyen, Hong Le Doan.

**Data curation:** Viet Anh Nguyen.

**Writing—original draft:** Viet Anh Nguyen, Thi Hanh Pham, Bich Ngoc Doan.

**Writing—review & editing:** Viet Anh Nguyen.
